# Pediatric Oncology

**DOI:** 10.1080/13577140120097085

**Published:** 2001-11


### PEDIATRIC ONCOLOGY

**105 Factor Receptor Associated with Decreased Metastases in Human Rhabdomyosarcoma Xenograft Model**

Fariba Navid[1], Kristi Lewis[1], Roger Abounader[2], John Laterra[2], Chand Khanna[1], Lee Helman[1]
*[1]National Cancer Institute, [2]Kennedy Krieger Institute*

*Objective:* The product of the met protooncogene, Met, is a tyrosine kinase receptor for hepatocyte growth factor (HGF). This signaling pathway has been shown to be important in a number of normal as well as malignant cellular processes including proliferation, metastases, motility, morphogenesis, and angiogenesis. The overexpression of Met has been reported in a number of sarcomas including rhabdomyosarcoma. We were interested in elucidating the role of Met overexpression in rhabdomyosarcoma.
*Methods:* In order to gain a better understanding of the role of Met overexpression in rhabdomyosarcoma, we attempted to inhibit the expression of Met by transfecting an embryonal rhabdomyosarcoma cell line, RD4A, with a met antisense construct and a chimeric U1 small nuclear RNA ribozyme antisense construct. The biologic consequence of orthotopic as well as tail vein injections of stable clones obtained using these constructs was studied in SCID/Beige mice.
*Results:* Using the ribozyme construct, we were successful at maintaining one stable clone with an approximately 70% decrease in Met expression as compared to the mock transfected controls. Similarly, one stable clone with a 50% decrease in Met protein expression as compared to the mock transfected clone was isolated from the met antisense transfectants. Several of the stable clones with significant knock down of Met expression could not be propagated. A preliminary in vivo experiment using the met ribozyme clone and controls in SCID/Beige mice was conducted to assess the affects of down regulation of Met in the establishment and growth of tumor in the gastrocnemius muscle. No significant difference in time to tumor take or tumor growth was observed in this small cohort of mice. When the same cell lines were injected via tail vein, none of the mice that received the cell line transfected with the met ribozyme construct developed tumor metastases. Based on these preliminary observations, we expanded the cohort of mice used in this experiment. In addition, we included the RD4a clone transfected with the met antisense clone to make sure our observations were not due to clonal variability within our cell line. Fifty days post tail vein injection, 7 of 10 mice injected with the mock transfected ribozyme control cell lines had evidence of metastatic disease whereas 0 of 10 mice injected with the met ribozyme knock down clone had evidence of metastatic disease. Similarly, 6 of 10 mice injected with the mock transfected control had evidence of tumor metastases whereas 1 of 10 mice injected with antisense transfected clone had evidence of tumor metastases 43 days post injection. Taking into consideration the sequential tail vein experiments, 23 of 30 mice injected with tumor cells containing wild type Met developed metastases and 1 of 25 mice with clones expressing decreased Met developed metastases. Using Fisher's exact t-test, this data is extremely significant with a p-value of less than 0.0001.
*Conclusion:* These observations would suggest that Met plays a role in the metastatic process in rhabdomyosarcoma and may serve as an excellent target in the setting of minimal residual disease.

DOI: 10.1080/13577140120097085

